# A Cleared View on Retinal Organoids

**DOI:** 10.3390/cells8050391

**Published:** 2019-04-28

**Authors:** Virginia Cora, Jasmin Haderspeck, Lena Antkowiak, Ulrich Mattheus, Peter H. Neckel, Andreas F. Mack, Sylvia Bolz, Marius Ueffing, Natalia Pashkovskaia, Kevin Achberger, Stefan Liebau

**Affiliations:** 1 Institute of Neuroanatomy & Developmental Biology (INDB), Eberhard Karls University Tübingen, Österbergstrasse 3, 72074 Tübingen, Germany; virginia.cora@uni-tuebingen.de (V.C.); jasminhaderspeck@web.de (J.H.); lena.antkowiak@uni-tuebingen.de (L.A.); natalia.pashkovskaia@mailbox.tu-dresden.de (N.P.); 2 Institute of Clinical Anatomy and Cell Analysis, University of Tübingen, 72074 Tübingen, Germany; ulrich.mattheus@uni-tuebingen.de (U.M.); peter.neckel@uni-tuebingen.de (P.H.N.); an.mack@uni-tuebingen.de (A.F.M.); 3 Centre for Ophthalmology, Institute for Ophthalmic Research, University of Tübingen, 72076 Tübingen, Germany; sylvia.bolz@uni-tuebingen.de (S.B.); marius.ueffing@uni-tuebingen.de (M.U.); 4 Center for Neurosensory Systems (ZFN), Eberhard Karls University Tübingen, 72076 Tübingen, Germany

**Keywords:** retinal organoid, human iPSC, PACT, CLARITY, organoid

## Abstract

Human induced pluripotent stem cell (hiPSC)-derived organoids mimicking tissues and organs in vitro have advanced medical research, as they opened up new possibilities for in-depth basic research on human organ development as well as providing a human in vitro model for personalized therapeutic approaches. hiPSC-derived retinal organoids have proven to be of great value for modeling the human retina featuring a very similar cellular composition, layering, and functionality. The technically challenging imaging of three-dimensional structures such as retinal organoids has, however, raised the need for robust whole-organoid imaging techniques. To improve imaging of retinal organoids we optimized a passive clearing technique (PACT), which enables high-resolution visualization of fragile intra-tissue structures. Using cleared retinal organoids, we could greatly enhance the antibody labeling efficiency and depth of imaging at high resolution, thereby improving the three-dimensional microscopy output. In that course, we were able to identify the spatial morphological shape and organization of, e.g., photoreceptor cells and bipolar cell layers. Moreover, we used the synaptic protein CtBP2/Ribeye to visualize the interconnection points of photoreceptor and bipolar cells forming the retinal-specific ribbon synapses.

## 1. Introduction

In the past decade, personalized medicine approaches traversed major changes with the breakthrough discovery of human induced pluripotent stem cells (hiPSCs) [1]. The capability to reprogram adult somatic cells to a pluripotent state opened up new venues for the development of personalized therapeutic strategies and respective in vitro models. Although major progress was achieved by combining iPSC-technology and two-dimensional culture techniques, the missing three-dimensional multi-cellular organization still represented a huge limitation. Lately, this gap was partially filled by the development of the stem-cell derived three-dimensionally organized tissue, called organoids [2]. Organoids, because of their proximity of cell-type composition, structural organization and functionality to the respective in vivo tissue, are extremely attractive for a broad applicability ranging from the understanding the basic developmental dynamics to drug treatment personalization or autologous cell therapy [3,4,5].

Amongst existing organoid models such as brain [6], pancreas [7], or kidney [8] that have been developed during recent years, retinal organoids stand out given their amazing resemblance with the human retina. Already in the initial publications, the presence of all major retinal cell types, including photoreceptor cells, organized in a highly physiological manner, had been reported [9,10]. In addition, the formation of retina-specific structures like the ribbon synapse [11,12] and retinal cell-specific features such as the photoreceptors segments [10,12], were described. Even more astonishing was the observation that photoreceptor cells of retinal organoids have the capability to react to light stimuli and pass the information to the inner retina in a physiological manner [10,12].

This ability to create a functional and complex cellular network within the organoid created the need for robust labeling and imaging techniques to characterize cellular morphology as well as to study tissue organization and intra-system cellular interactions. Physical sectioning, as well as whole-mount staining techniques, have been applied to image retinal organoids in two- or three-dimensional immunocytochemistry [9,10,13,14]. These methods, however, have the disadvantage that they either cannot reflect the full three-dimensional morphology of the cells (physical sectioning) or they cannot be imaged with high resolution (whole-mount staining), mainly because of the low diffusion of molecular labels through the tissue barriers and limited depth of light penetration due to tissue light scattering [15]. These issues can be overcome by applying tissue clearing coupled with optical sectioning techniques as shown previously in the pioneering study of whole brain imaging [16]. Tissue clearing techniques are based on the concept of decreasing the inhomogeneities in light-scattering of tissue by matching the refractive index throughout the sample. This is achieved for example by removing membrane lipids, which are one of the main causes of light-scattering [15,17]. Among the techniques developed over time, those that couple solvent-based clearing with hydrogel embedding of the sample (e.g., passive clarity technique (PACT), perfusion-assisted agent release in situ (PARS), CLARITY) are particularly interesting for organoid clearing since they combine the possibility of clearing large samples without a significant loss of proteins during membrane dissipation [16,18].

Here we present an adapted version of the hydrogel embedding-based clearing approach called passive clarity technique (PACT) [19] for the imaging of whole retinal organoids. We show that by using PACT we can obtain more defined spatial information via optical sectioning methods such as light sheet microscopy. Moreover, we demonstrate the possibility to reconstruct and study the fine morphology of organoid cells in cleared tissues as well as interconnection points using the example of the photoreceptor ribbon synapses.

## 2. Materials and Methods

### 2.1. hiPSC Culture

hiPSC lines were derived from keratinocytes of healthy donors as previously described [20]. hiPSCs were maintained in FTDA medium [21] on tissue culture treated plates (BD Biosciences, Franklin Lakes, NJ, USA) coated with hESC-qualified Matrigel (BD Biosciences) at 5% O_2_ and 5% CO_2_ atmosphere with a constant temperature of 37 °C. Differentiating cells were mechanically removed by scraping. All the lines used in this study were tested for stem cell markers and germ-layer differentiation potential. All procedures were in accordance with the Helsinki convention and approved by the Ethical Committee of the Eberhard Karls University Tübingen (Nr. 678/2017BO2). Control persons gave their written consent.

### 2.2. hiPSCs-Derived Retinal Organoids Culture

The generation of hiPSC-derived retinal organoids was performed based on the procedure described by Zhong et al. [10] with some modifications. On day 0, embryoid bodies (EBs) were generated. First, hiPSC were dissociated to single cells using TrypLE (ThermoFisher Scientific, Waltham, MA, USA) and 2.88 × 10^6^ cells suspension was distributed into untreated v-shaped 96-wells (Sarstedt, Nümbrecht, Germany) in PeproGrow (PeproTech, Rocky Hill, NJ, USA) medium supplemented with 10 µM Y-27632 (ROCK-inhibitor, Ascent Scientific, Bristol, UK) and 10 µM blebbistatin (Sigma-Aldrich, St. Louis, MO, USA). Cells were re-aggregated by centrifugation at 400× *g* for 4 min. On day 1, 80% of the medium was removed and replaced with Neural Induction Medium (NIM) composed of DMEM/F12 (1:1) + Glutamax supplement (ThermoFisher Scientific), 24 nM sodium selenite (Sigma-Aldrich), 16 nM progesterone (Sigma-Aldrich), 80 µg/mL human holotransferrin (Serologicals, Norcross, GA, USA), 20 µg/mL human recombinant insulin (Sigma-Aldrich), 88 µM putrescin (Sigma-Aldrich), 1x minimum essential media-non essential amino acids (NEAA, ThermoFisher Scientific), 1x antibiotics-antimycotics (AA, ThermoFisher Scientific). The medium was changed one more time on day 4. The EBs were seeded at day 7 on 6 well plates coated with growth-factor-reduced matrigel (BD Biosciences) with a density of 32 EBs/well. Medium change was performed daily. At day 16 the NIM medium was substituted with B27-based differentiation medium (BRDM) composed of DMEM/F12 (3:1) supplemented with 2% B27 (*w*/*o* vitamin A, ThermoFisher Scientific), 1x NEAA and 1x AA. The neural retina fields were lifted using 10 µL tips on day 24. The detached areas were then collected in 10 cm bacterial petri dishes (Greiner Bio-One, Kremsmünster, Austria) and harvested in suspension in the BRDM. For the first day after detachment BRDM medium was supplemented with 10 µM ROCK-Inhibitor Y-27632. Over the following weeks all non-retinal vesicles were discarded and, using micro scissors, the non-retinal portions were mechanically excised from the retinal organoids. From day 40 onwards, BRDM was supplemented with 10% fetal bovine serum (FBS, Thermo Fisher Scientific) and 100 µM taurine (Sigma-Aldrich). From day 70–100, BRDM with FBS and taurine was further supplemented with 1 µM retinoic acid (Sigma-Aldrich), which was reduced to 0.5 µM during days 100–190 and removed afterwards. All the differentiation steps were cultured at 37 °C, 20% O_2_, and 5% CO_2_.

### 2.3. Lentiviral Transfection of Retinal Organoids

Retinal organoids (day 155) were transfected with a lentiviral vector expressing enhanced green fluorescent protein (eGFP) under the interphotoreceptor retinoid binding protein (IRBP) promoter for further studies. The vector labeling photoreceptor cells was provided as a gift from Deepak Lamba & Thomas Reh (University of Washington, Washington, DC, USA) [22]. To improve transfection efficiency, 8 µg/mL Polybrene ^®^ (Sigma Aldrich) were added to the lentivirus. Incubation was performed overnight and organoids were afterwards washed with BRDM.

### 2.4. Tissue Clearing

Retinal organoids were fixed overnight at 4 °C in the hydrogel monomer solution (HMS) composed of 4% paraformaldehyde (PFA) (AppliChem GmbH, Darmstadt, Germany), 5% acrylamide solution (AppliChem GmbH) supplemented with 0.25% of 2,2’-Azobis [2-(2-imidazolin-2-yl) propane] Dihydrochloride initiator (VA-044, FUJIFILM WAKO Chemicals GmbH, Richmond, VA, USA) in Dulbecco’s phosphate-buffered saline (PBS, no calcium, no magnesium, Thermo Fisher Scientific). Each HMS infused sample was then placed into a 500 μL tube (Eppendorf, Hamburg, Germany) and covered completely with fresh HMS. The samples were degassed at 13.3 kPa for 30 min using a vacuum oven (Thermo Scientific) and the sample-hydrogel hybridization was achieved by heating the samples at 45–50 °C for 2 h. After the polymerization was completed, each sample was cut out from the hydrogel matrix and transferred into 2 mL tube (Eppendorf) containing 8% sodium dodecyl sulphate (AppliChem GmbH) in PBS at pH 7.4. Samples were then incubated under continuous rotation at 45 °C for 5 days. Before immunocytochemistry, cleared samples were washed three times over the course of the day using PBS.

### 2.5. Immunocytochemistry

For whole-mount immunocytochemistry of cleared and control uncleared PFA-fixed retinal organoids, blocking and permeabilization was performed overnight at 37 °C using a solution of 10% normal donkey serum (Merck Millipore, Burlington, MA, USA), 0.2% Triton X-100 (Carl Roth, Karlsruhe, Germany), and 1x AA in PBS. Primary antibodies were diluted in blocking solution and incubated with the samples for 4 days at 37 °C. Samples were then washed 3–4 times at 37 °C using PBS with 0.2% Triton X-100. Then, secondary antibodies were diluted in a solution of 5% normal donkey serum, 0.1% Triton X-100, and 1x AA and applied to the samples for 4 days at 37 °C. Next, samples were incubated with DAPI for 1 h at 37 °C in PBS with 0.2% Triton X-100. Finally, samples were washed as after primary antibodies incubation and one more time with PBS alone. Mounting was performed by incubation of the cleared samples in 80% glycerol (Carl Roth) in dH_2_O until the specimens were fully equilibrated with the solution and therefore entirely transparent. All staining steps as well as the mounting were performed with gentle shaking.

For cryosections immunocytochemistry, first cleared and control uncleared PFA-fixed retinal organoids were immersed in 30% sucrose (Sigma-Aldrich) overnight until the samples were completely equilibrated with the medium. Following, the samples were embedded in cryo molds using Tissue-Tek O.C.T. (Sakura Finetek, Flemingweg, Netherlands) and sectioned using a cryostat. After, the cryosections were rehydrated for 30 min in PBS, blocking and permeabilization was performed using 10% normal donkey serum with 0.2% triton-X in PBS for 1 h at room temperature. Primary antibodies were diluted in the blocking solution and applied overnight at 4 °C to the samples. The sections were then washed three times with PBS before proceeding. The secondary antibodies were diluted in a solution of 5% normal donkey serum with 0.1% Triton X-100 in PBS and incubated with the samples for 1 h at room temperature. The sections were washed again three times with PBS and mounted using ProLong Gold Antifade Reagent with DAPI (Thermo Fisher Scientific).

- Antibodies used in immunocytochemistry:
- Primary antibodies:
- Arrestin 3 (1:100, sc54355, Santa Cruz Biotechnologies, Dallas, TX, USA)
- CRALBP [B2] (1:250, ab15051, Abcam, Cambridge, UK)
- CtBP2 (Ribeye) (1:200, 612044, BD Biosciences)
- Glutamine Synthase (1:500, MAB302, Merck Millipore)
- Opsin, blue (1:200, AB5407, Merck Millipore)
- PKCα (1:500, sc208, Santa Cruz Biotechnologies)
- Recoverin (1:1000, sc20353, Santa Cruz Biotechnologies)
- Rhodopsin (1:200, sc57432, Santa Cruz Biotechnologies)
- ROM1 (1:200, 21984-1-AP, Proteintech, Rosemont, IL, USA)
- ZO-1 (1:100, 33-9100, Thermo Fisher Scientific, USA)
- Secondary antibodies:
  - Donkey anti-Mouse Alexa Fluor^®^ 488/568 (1:250, Thermo Fisher Scientific)
  - Donkey anti-Rabbit IgG (H + L) Alexa Fluor^®^ 488/568 (1:250, Thermo Fisher Scientific)
  - Donkey anti-Goat IgG (H + L) Alexa Fluor^®^ 647 (1:250, Thermo Fisher Scientific)
- Antibodies used in whole-mount immunocytochemistry:
- Primary antibodies:
  - Arrestin 3 (1:50, sc54355, Santa Cruz Biotechnologies)
  - CtBP2 (Ribeye) (1:100, 612044, BD Biosciences)
  - GFP (1:500, A-6455, Thermo Fisher Scientific)
  - PKCα (1:250, sc208, Santa Cruz Biotechnologies)
  - Recoverin (1:500, sc20353, Santa Cruz Biotechnologies)
  - Rhodopsin (1:100, sc57432, Santa Cruz Biotechnologies)
  - β-III-Tubulin (1:200, 802001, BioLegend, San Diego, CA, USA)
- Secondary antibodies:
  - Donkey anti-Mouse Alexa Fluor^®^ 488/568/647 (1:1000, Thermo Fisher Scientific)
  - Donkey anti-Rabbit IgG (H + L) Alexa Fluor^®^ 488/568/647 (1:1000, Thermo Fisher Scientific)
  - Donkey anti-Goat IgG (H + L) Alexa Fluor^®^ 488/568/647 (1:1000, Thermo Fisher Scientific)

### 2.6. Fluorescence Microscopy

Retinal organoids cryosections were analyzed using a conventional fluorescent microscope and image stacks were acquired using an Imager M2 Apotome1 (Carl Zeiss, Oberkochen, Germany). For whole cleared retinal organoids analysis, each sample was glued to a glass capillary, submerged in the sample chamber filled with 80% glycerol in H_2_O (refracting index 1.45) and imaged using a light sheet microscope (Lightsheet, Carl Zeiss). Images were analyzed, processed and 3Drendered using ZEN 2.3 Blue Edition (Carl Zeiss).

### 2.7. Electron Microscopy

Electron Microscopy was performed as previously described [23].

## 3. Results

### 3.1. Passive Clarity Technique (PACT) Protocol Optimized for the Retinal Organoid

**Figure 1:**
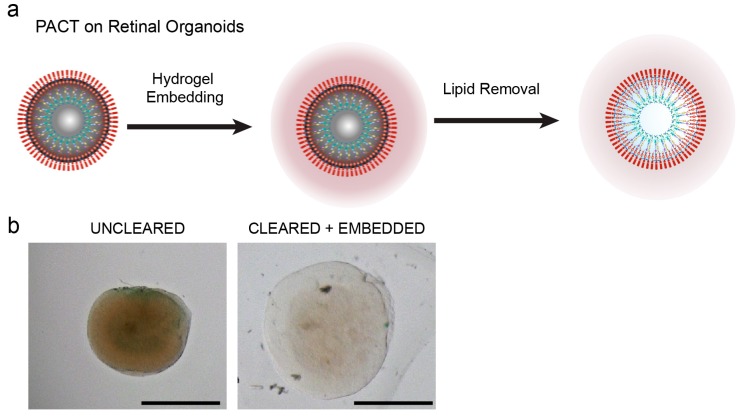
Passive clarity technique (PACT) protocol optimized for the retinal organoid. (**a**) Schematic workflow of retinal organoid-clearing with PACT. Initially, organoids (left) are embedded in a hydrogel (middle). Membrane lipids are washed away using a detergent solution to finally obtain an optically transparent sample (right). (**b**) Comparison of an uncleared retinal organoid (day 106) with a hydrogel-embedded organoid cleared for 5 days using optimized PACT. Both organoids are immersed in 80% glycerol. Scale bars: (**b**) 500 µm.

In order to improve the imaging of three-dimensional retinal organoids, we established and optimized a clearing protocol based on the passive clarity technique (PACT) [18,19]. A schematic protocol of the workflow is illustrated in Figure 1a. First, paraformaldehyde-fixed retinal organoids were infused with a hydrogel monomer solution that was polymerized at a temperature of 45–50 °C. This led to the formation of an organoid–hydrogel hybrid (Figure 1a) to which the proteins of the tissue were anchored preventing them to be washed away during the next step. Hydrogel embedded organoids were subsequently submerged into an amphiphilic compound to passively remove membranes lipids. After one week of clearing at 50 °C, retinal organoids appeared highly transparent in comparison to uncleared ones (Figure 1b).

### 3.2. PACT Greatly Improves Retinal Organoid Immunocytochemistry and High-Resolution Imaging

**Figure 2:**
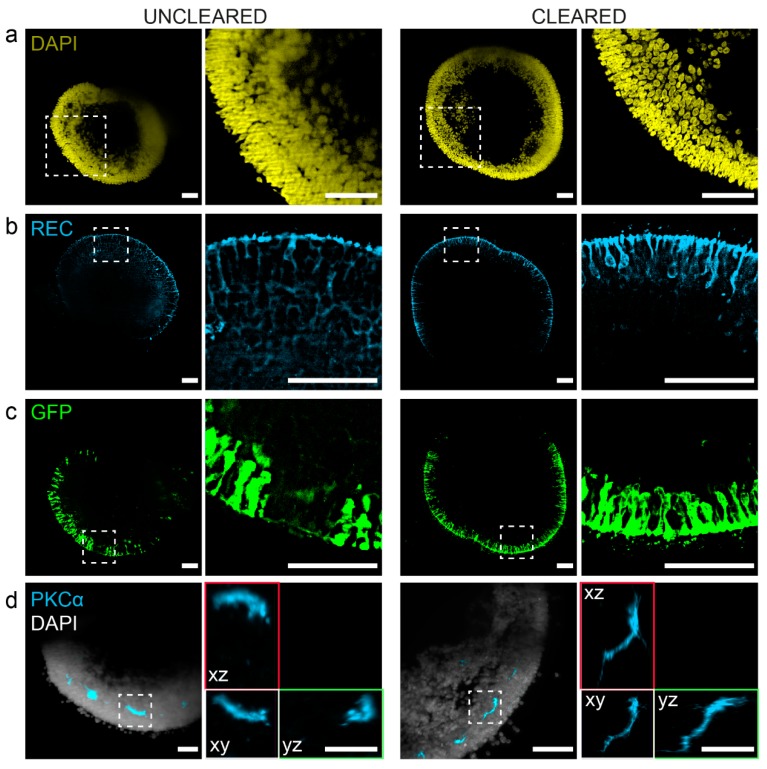
PACT greatly improves retinal organoid immunocytochemistry and high-resolution imaging. Retinal organoids were immunostained and imaged by light sheet microscopy. Single planes were selected from an acquired image stack to visualize differences between uncleared (left panel) and cleared (right panel) retinal organoids. Each panel contains an overview (left image) and magnified cutout (right image). Organoids were labeled as followed: (**a**) day 182 retinal organoids were labeled with DAPI (4′,6-Diamidin-2-phenylindol, nuclei staining, yellow). Second and forth image show a magnified area as indicated in the first and third image, respectively. (**b**) Day 182 retinal organoids were stained for Recoverin (pan-photoreceptor marker). Second and forth image show a magnified area as indicated in the first and third image, respectively. (**c**) Day 182 retinal organoids were previously transfected with a lentiviral vector labeling photoreceptor cells (pJG-IRPB-eGFP). Cleared or uncleared retinal organoids were additionally stained by a GFP-antibody. (**d**) Day 290 retinal organoids were stained for PKCα (bipolar cell marker, light blue) and DAPI (nuclei, white). Second and forth image show xy-, xz-, and yz-plane views of a magnified bipolar cell. Scale bars: (**a**–**c**) 100 µm, (**d**) 50 µm.

In order to validate the benefits of cleared retinal organoids in comparison to uncleared, we compared the possibility to perform whole mount immunocytochemistry and high-resolution imaging using light sheet microscopy (Figure 2). A list of tested working and not-working antibodies is provided (Table S1). Exemplary immunostainings of retinal markers on retinal organoids are given in Figure S1. Using the small fluorescent molecule 4′,6-Diamidin-2-phenylindol (DAPI), we first evaluated the ability of small molecules to enter the organoid tissue. Comparing single images of a whole organoid light sheet stack, we can demonstrate that both cleared and uncleared organoids allow thorough penetration of DAPI into the tissue (Figure 2a). Although the DAPI signal was sharp at the surface of the uncleared organoid, the inner layers appeared blurred, whereas the imaging quality of DAPI in cleared organoid was sharp throughout all layers. Next, we tested immunocytochemistry using the bona fide pan-photoreceptor marker Recoverin (Figure 2b). Here, cleared retinal organoids showed a strong and clear staining of photoreceptors in the outer retinal organoid layers, whereas the Recoverin staining of uncleared retinal organoids could not be well separated from background. Therefore, we can assume that labeling and penetration by antibodies seems to be improved in cleared retinal organoids.

Next, we wanted to validate if enhanced green fluorescent protein (eGFP) is preserved during the clearing process. For that, we transduced retinal organoids with lentivirus carrying eGFP under the control of a photoreceptor-specific promoter IRBP (Interphotoreceptor retinoid-binding protein). After the transfected organoids were processed, we stained the transfected organoids with an eGFP-antibody. After light sheet imaging, in both uncleared and cleared organoids it was possible to detect eGFP using a GFP antibody (Figure 2c).

Next, we evaluated whether imaging was also improved in the inner parts of the retinal organoids. Using the bipolar cell marker PCKα (Protein kinase C alpha), we could visualize the fine soma and process structure in a high signal quality in all three dimensions (Figure 2d). In comparison, labeled bipolar cells of uncleared retinal organoids remained blurred and undetailed. Using the neuron marker β-III-Tubulin (TUJ), we could even visualize the delicate neural meshwork in the innermost of cleared retinal organoids (Figure S2, Movie S1).

In summary, PACT clearing of retinal organoids largely increases the antibody penetration and specificity. In combination with reduced light scattering, this improves the quality of three-dimensional immunocytochemistry and imaging of whole organoids.

### 3.3. Visualization of Photoreceptor Morphology

**Figure 3:**
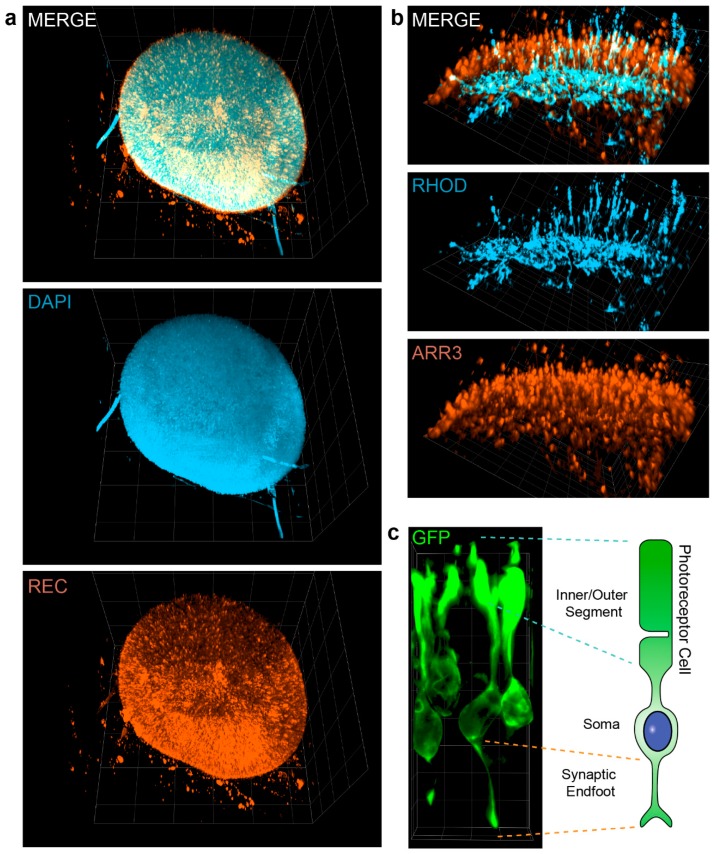
Visualization of photoreceptor morphology. (**a**) Three-dimensional rendered image of a cleared whole retinal organoid (day 182) based on a light sheet image stack. Retinal organoids were stained for DAPI (nuclei staining, light blue) and Recoverin (REC, pan-photoreceptor marker, orange). (**b**) Three-dimensional rendered image of a day 260 cleared retinal organoid stained for the rod marker Rhodopsin (RHOD, light blue) and the cone marker Arrestin 3 (ARR3, orange). (**c**) Day 182 cleared retinal organoid transfected with a lentiviral vector labeling photoreceptor cells (pJG-IRPB-eGFP) and counterstained by a GFP-antibody. Image was 3D rendered from a small stack that was excised from a light sheet-acquired image. Grid square side length in rendered 3D images: (**a**) 200 µm, (**b**) 40 µm, and (**c**) 9.3 µm.

The photoreceptor cell is fundamentally important for the functionality of the retina by detecting and processing light information. This ability demands for specialized and unique structures such as the photoreceptor inner and outer segments and the synaptic endfeet containing the ribbon synapse (See also Figure 3c). The use of PACT clearing allows the study of the three-dimensionally photoreceptor morphology. Using the pan-photoreceptor marker Recoverin, the whole photoreceptor population of an organoid can be visualized by light sheet microscopy (Figure 3a, Movie S2). Using cell-type specific markers, it is also possible to distinguish the two major photoreceptor subtypes, rods and cones (Figure 2b and Movie S3). Similar to the in vivo situation, rods stained for Rhodopsin show a more inner localization, while cones are oriented more outside of the organoid. Finally, we visualized a group of photoreceptor cells in detail demonstrating the high-resolution quality of the cleared tissue (Figure 3c and Movie S4). Similar to the schematic make-up (Figure 3c, right), retinal organoid photoreceptors possess a basal synaptic endfoot and an apical two-parted segment-like structure. In summary, PACT clearing of retinal organoids enables the visualization of the complete photoreceptor population down to the morphology of the single photoreceptor.

### 3.4. Visualization of the Photoreceptor Ribbon Synapse

**Figure 4:**
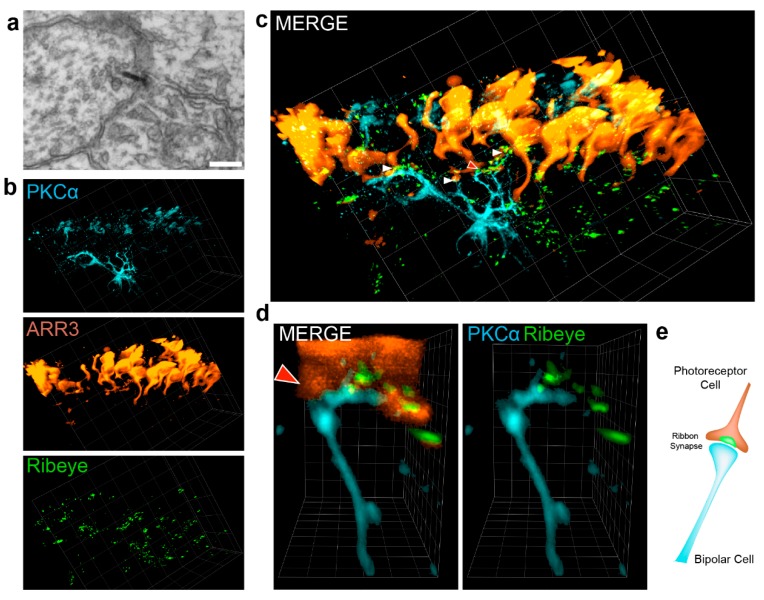
Visualization of the photoreceptor ribbon synapse. (**a**) Ribbon synapse identified in electron microscope from day 196 retinal organoids. (**b**–**d**) Light sheet microscopy of the ribbon synapse. Retinal organoids of day 290 were cleared and stained for the cone photoreceptor marker Arrestin 3 (ARR3, orange), the bipolar cell marker PKCα (light blue) and the ribbon synapse marker Ribeye (CtBP2, green). White Arrows indicate ribbon synapse. Red Arrow with white outline points to ribbon synapse imaged magnified in d). (**e**) Schematic depiction of the photoreceptor ribbon synapse formed with a bipolar cell found in retinal organoids as shown in (**d**). Grid square side length in rendered 3D images: (**b**–**c**) 20 µm and (**d**) 2.6 µm.

To show the potential of PACT clearing, we visualized ribbon synapses of retinal organoids in three dimensions. In the retina, ribbon synapses are formed between photoreceptors endfoot-invaginations and bipolar cells together with horizontal cells. The ultrastructure is characterized by the presence of an electron-dense structure, called synaptic ribbon, where synaptic vesicles are held in place near the active zone. In retinal organoids, these structures can be identified upon electron microscopy analysis (Figure 4a) and by specific immunostaining targeting the ribbon protein Ribeye, an isoform of CtBP2. Using PACT cleared organoids, the network of ribbon synapses formed by photoreceptor and bipolar cells can be imaged (Figure 4b–d). Visualizing the fine network of bipolar dendrites (PKCα-positive), we successfully identified multiple connection points with photoreceptor endfeet labeled by Arrestin 3 (arrowheads in Figure 4c, Movie S5). Co-labeling with Ribeye verified the ribbon nature of the synapse (Figure 4b–c, green). In Figure 4d, one ribbon synapse was exemplarily magnified. Here, the Ribeye staining highlights the interconnecting points between bipolar dendrites and the photoreceptor axon. Moreover, it verified that indeed the ribbon signal is restricted to the presynaptic photoreceptor endfoot (Figure 4d, see also Movie S6). Taken together, PACT is a powerful tool for imaging the three-dimensional network of neuronal cells, their processes, and synaptic connections.

## 4. Discussion

The complexity of three-dimensional tissue structures (especially in the context of the nervous system) challenged imaging techniques and was one of the major driving forces for the development of a new generation of tissue clearing methods with the pioneering method called CLARITY [16]. This novel procedure allows to obtain enhanced information about cellular morphology, three-dimensional tissue composition, and cell-cell interconnections, and is therefore of great impact for neurobiological studies.

In this study, we provide for the first time a sustainable protocol for the successful tissue clearing of retinal organoids that increases the quality of whole-sample imaging. We used the hydrogel-based passive clearing technique (PACT) on retinal organoids and improved the quality of three-dimensional imaging upon immunocytochemistry staining compared to classical approaches (e.g., whole-mount). PACT provides an easy way of clearing retinal organoids by passively removing membrane lipids, which represent the major obstacle in the imaging of three-dimensional structures due to light-scattering. Moreover, PACT is a hydrogel-based clearing method that has been shown to be compatible with immunocytochemistry approaches [16,18]. This approach is based on the infusion of the tissue with hydrogel monomers that can be polymerized and form a tissue-hydrogel hybrid. Consequently, the biomolecules are either covalently anchored to or physically immobilized in the hydrogel mesh while the lipids are washed out. The tissue becomes then accessible to molecular labels and optically transparent allowing an in-depth interrogation of the sample by keeping intact structural and molecular information.

Although tissue clearing is known to disrupt some epitopes, especially those that are located in the membrane due to membrane lipid wash-out, we show that numerous epitopes are left intact after the clearing procedure. To support further studies, we provide here a list of antibodies that we tested on cleared retinal organoid sections, targeting commonly used epitopes such as cell population markers in retinal research (Figure S1 and Table S1). Moreover, we demonstrate that the fluorescent reporter GFP is stable during the clearing procedure and provides structural information more detailed than any other marker used. Similar results have been shown in previous studies [16,18,24], in which GFP reporters facilitate the interrogation of cleared samples because of the robustness of the signal, making GFP reporter a perfect tool to be combined with tissue clearing approaches.

Even though immunocytochemistry plays a crucial role in the characterization of retinal organoids, other imaging approaches can be of interest, such as electron microscopy methods. It has been already shown that PACT is a gentler approach compared to other tissue clearing methods and the ultrastructural details are relatively well preserved [19]. This allows pursuing further investigations on the cleared samples such as electron microscopy analysis.

Lately, retinal organoid technology has emerged as one of the most promising tools for modeling the human retina, especially with respect to disease pathology or drug side-effect evaluation [25]. Three-dimensional cell branching, the presence of synaptic interconnections and the layered organization of cell populations are crucial features that can be used to evaluate the condition of the tissue. Thus, the enhanced visualization of tissue details provides a powerful tool for the organoid research field. Drug toxicity tests, for example, require a comprehensive view of changes in the system, while for neurodevelopmental disease models it is crucial to be able to extensively characterize cell population dynamics.

In this study, we demonstrate how tissue-clearing techniques such as PACT could be beneficial for retinal or general organoid research, in addition to the classical physical sectioning approach, which leads to the loss of three-dimensional information, or whole mount staining, which is limited by the low tissue penetration of molecular labels as well as by the light-scattering. Moreover, since dynamics in the retinal tissue development in human are still widely unclear, the possibility to apply several lineage-tracing techniques such as the confetti approach [26] coupled with the possibility to gain intact tissue information provided by clearing methods could be of great help for answering basic developmental questions.

## Supplementary Materials

The following are available online at https://zenodo.org/record/2653063#.XMWELKLYq-M, Figure S1, Table S1, Videos S1–S6.
